# Unmasking Focal Segmental Glomerulosclerosis in a Patient With Lupus Nephritis

**DOI:** 10.7759/cureus.63008

**Published:** 2024-06-24

**Authors:** Talar Acob, Omar Jumaah, Tigran Kakhktsyan, Knkush Hakobyan, Sajina Prabhakaran

**Affiliations:** 1 Internal Medicine Residency Program, Capital Health Regional Medical Center, Trenton, USA; 2 Internal Medicine, Capital Health System, Trenton, USA; 3 Rheumatology, Capital Health Regional Medical Center, Trenton, USA

**Keywords:** focal segmental glomerular sclerosis, nephrotic range proteinuria, systemic lupus erythematosus disease, biopsy proven renal disease, sle and lupus nephritis

## Abstract

Systemic lupus erythematosus (SLE) is a persistent autoimmune disease where the immune system produces autoantibodies against nuclear and cytoplasmic antigens, leading to multi-organ involvement. Typically, nephrotic-range proteinuria is indicative of proliferative or membranous lupus nephritis. However, on rare occasions, patients with SLE and nephrotic syndrome may exhibit focal segmental glomerulosclerosis (FSGS) lesions upon kidney biopsy. We describe a 28-year-old Middle Eastern male with SLE and biopsy-proven lupus nephritis who experienced a significant increase in proteinuria and creatinine levels despite treatment with mycophenolate mofetil. Further investigation revealed FSGS without active lupus nephritis. The patient's treatment regimen was adjusted to rituximab in response to these findings. This case underscores the importance of renal biopsies in SLE management to accurately diagnose and tailor treatment. It highlights the need to consider alternative renal complications, such as FSGS, which can coexist with lupus nephritis, necessitating a collaborative approach between rheumatologists and nephrologists to achieve optimal patient outcomes.

## Introduction

Systemic lupus erythematosus (SLE) is a chronic autoimmune disorder marked by the production of autoantibodies targeting nuclear and cytoplasmic antigens, impacting multiple organs [1]. Nephrotic-range proteinuria often indicates proliferative lupus nephritis and/or membranous lupus nephritis. However, in rare cases (3-10%), SLE patients with nephrotic syndrome may exhibit kidney biopsy results showing focal segmental glomerulosclerosis (FSGS) lesions [2]. This case report discusses the rare occurrence of FSGS alongside lupus nephritis in a patient with SLE.

This article was previously presented as a poster at the American College of Physicians (ACP) New Jersey Meeting on March 8, 2024, the Society of Hospital Medicine Meeting on April 13, 2024, and the Congress of Clinical Rheumatology on May 8, 2024.

## Case presentation

A 28-year-old Middle Eastern male, previously diagnosed with SLE and biopsy-proven lupus nephritis at the age of 18 in Egypt, had been managing his condition with a daily dose of mycophenolate mofetil (MMF) at 250 mg. During a routine clinic follow-up, laboratory tests were conducted, revealing concerning results. His 24-hour urine protein level had risen to a significant 2,202 mg/dL, and his creatinine levels had increased to 2.8 mg/dL from a baseline of 1.12 mg/dL. In response to these alarming findings, the patient's treatment plan was adjusted, and the dose of MMF was increased to 500 mg daily. However, much to the medical team's surprise, despite the escalation of treatment, the 24-hour urine protein levels continued to climb, ultimately reaching 2,394 mg/dL. In addition to this, the patient displayed persistent normal C3 and C4 levels, negative results on double-stranded DNA testing, anti-Smith antibodies measuring less than 0.2 U/mL, and anti-chromatin antibodies at a level of 0.2 antibody index (AI) (Table 1).

**Table 1:** Laboratory findings AI, antibody index; anti-dsDNA, anti-double-stranded DNA; C3, complement factor 3; C4, complement factor 4

Test	Result	Normal range
C3	137 mg/dL	82-167 mg/dL
C4	36 mg/dL	12-38 mg/dL
Anti-dsDNA	22 IU/mL	0-9 IU/mL
Anti-Smith antibody	0.2 U/mL	0.0-0.9 AI
Serum creatinine level	2.8 mg/dL	0.76-1.27
Anti-chromatin antibody	<0.2 AI	0-9 AI
24-hour urine protein	2,394 mg/24 hours	30-150 mg/24 hours

**Figure 1:**
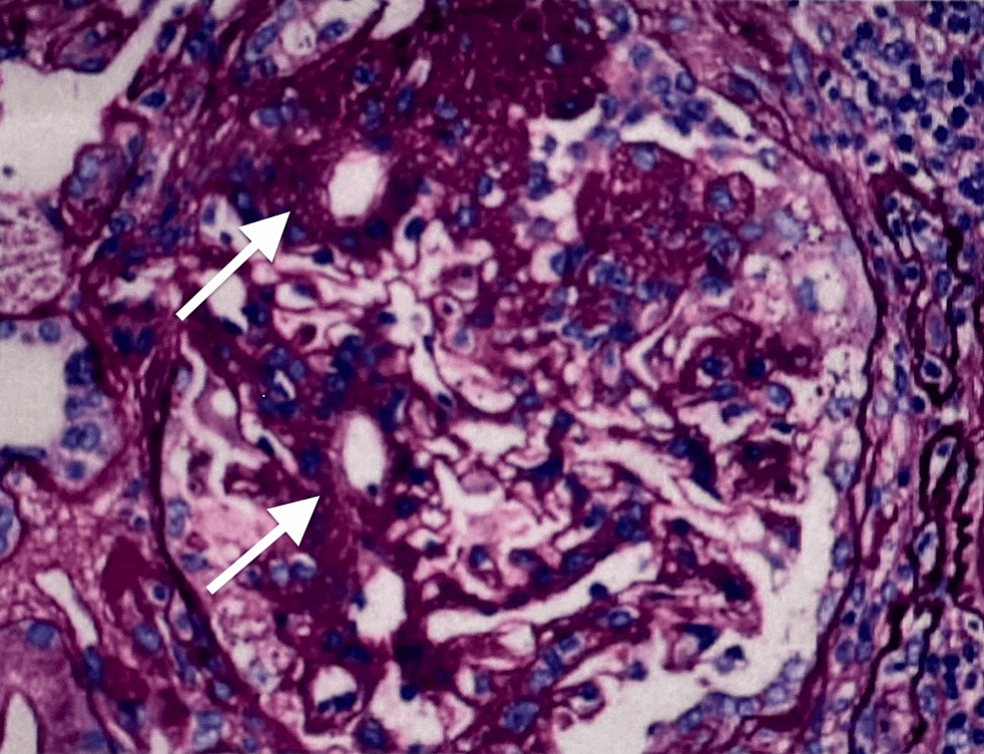
Biopsy of renal glomeruli showing segmental glomerulosclerosis (white arrows)

**Figure 2:**
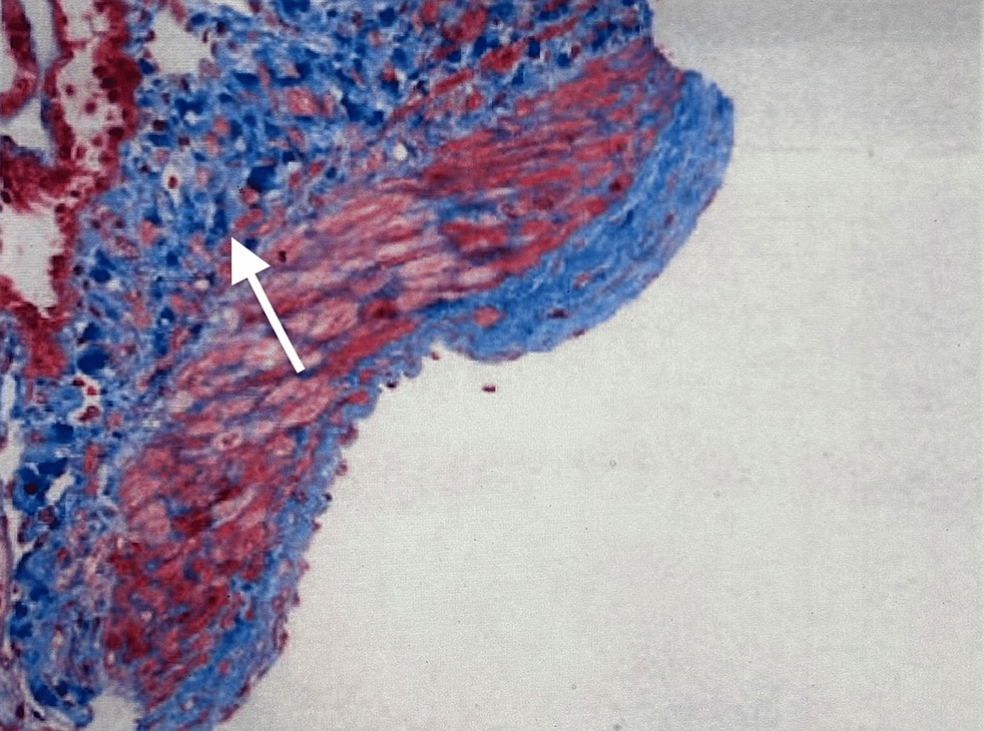
Renal biopsy showing intimal fibrosis (white arrow)

In light of these perplexing findings, a kidney biopsy was deemed necessary to gain further insights into the underlying renal condition. The biopsy results were unexpected; they revealed concurrent FSGS with no evidence of active lupus nephritis (Figures 1-2), challenging the initial diagnosis and treatment approach.

Subsequently, the medical team decided to wean the patient off MMF as the patient did not tolerate a higher dose of the medication (1,500 mg daily) due to adverse effects (headaches and muscle pain) and was started on IV rituximab 10 mg/mL. On a follow-up visit, the patient's lab results remained stable, with a serum creatinine ranging between 2.4 and 2.5 mg/dL. In addition, the patient remained clinically stable.

## Discussion

SLE is a chronic autoimmune disease characterized by widespread inflammation and tissue damage in various organs [3]. It is defined by the presence of autoantibodies and a broad spectrum of clinical manifestations that result from immune complex deposition and complement activation. The precise etiology of SLE is multifactorial, involving genetic, environmental, hormonal, and immunological factors. SLE predominantly affects women, with a female-to-male ratio of approximately 9:1. The disease commonly presents between the ages of 15 and 45, although it can occur at any age. Epidemiological studies indicate a higher prevalence among individuals of African, Hispanic, and Asian descent compared to those of European ancestry [1]. The overall prevalence of SLE varies geographically, with rates ranging from 20 to 150 cases per 100,000 individuals. SLE is a multisystem disease with a wide range of clinical manifestations. Common symptoms include fatigue, fever, arthralgia, and myalgia. The disease can affect almost any organ system, leading to diverse presentations: the musculoskeletal system (arthritis and arthralgia in over 90% of patients), the skin (malar rash, discoid rash, photosensitivity, and alopecia), the hematological system (anemia, leukopenia, lymphopenia, and thrombocytopenia), the cardiovascular system (pericarditis and myocarditis, increased risk of atherosclerosis), the nervous system (seizures, psychosis, and cognitive dysfunction), and the renal system, with lupus nephritis being one of the most serious complications. Renal involvement occurs in approximately 50-60% of SLE patients and significantly impacts morbidity and mortality. Lupus nephritis is categorized into six classes based on the International Society of Nephrology/Renal Pathology Society (ISN/RPS) classification: Class I (minimal mesangial lupus nephritis), Class II (mesangial proliferative lupus nephritis), Class III (focal lupus nephritis), Class IV (diffuse lupus nephritis), Class V (membranous lupus nephritis), and Class VI (advanced sclerosing lupus nephritis). Class IV is the most common and severe form, often leading to chronic kidney disease (CKD) and end-stage renal disease (ESRD) [4]. Patients with lupus nephritis typically present with proteinuria, hematuria, and varying degrees of renal impairment. Laboratory findings often include elevated serum creatinine, decreased glomerular filtration rate (GFR), and the presence of anti-double stranded DNA (anti-dsDNA) antibodies. Low complement levels (C3, C4) are also indicative of active disease. FSGS is a histopathological lesion that can be seen in SLE and lupus nephritis. It is characterized by scarring (sclerosis) within segments of some glomeruli. FSGS can occur as a primary condition or secondary to other diseases, including SLE. ACTN4 protein produced by renal podocytes is essential for maintaining the structural integrity of the glomeruli. Mutations in the ACTN4 protein weaken the podocytes, leading to the development of FSGS, where the kidney's filtering units scar and fail, causing the protein to leak into the urine. The prevalence of FSGS in lupus nephritis varies, but it is recognized as a potential pattern of glomerular injury in SLE patients. FSGS in SLE is associated with nephrotic syndrome and poorer renal outcomes [5]. The diagnosis of lupus nephritis and FSGS relies on clinical, serological, and histological evaluations, including clinical assessment (monitoring for signs and symptoms of renal involvement and regular laboratory tests for kidney function), serological tests (measurement of anti-dsDNA antibodies, complement levels, and other autoantibodies), and renal biopsy. A biopsy is a critical tool for diagnosing lupus nephritis and FSGS, with findings that guide classification and management. Histological examination reveals the extent and pattern of glomerular involvement, degree of activity (inflammation), and chronicity (scarring) [6]. In lupus nephritis, biopsy findings include mesangial proliferation, endocapillary proliferation, and immune complex deposits identified by immunofluorescence [7]. In FSGS, biopsy shows segmental areas of sclerosis within glomeruli, often with podocyte foot process effacement seen on electron microscopy [8]. The treatment of lupus nephritis involves immunosuppressive therapy to control inflammation and prevent progression to ESRD, including induction therapy with high-dose corticosteroids combined with either cyclophosphamide or MMF, maintenance therapy with lower doses of corticosteroids along with MMF or azathioprine to maintain remission, biologics like rituximab and belimumab in refractory cases, and supportive care with blood pressure control (angiotensin-converting enzyme inhibitors (ACEIs) or angiotensin II receptor blockers (ARBs)), lipid-lowering agents, and antimalarials like hydroxychloroquine. The management of FSGS in SLE follows similar principles but may also include calcineurin inhibitors (e.g., tacrolimus) and specific measures to address nephrotic syndrome. The prognosis of lupus nephritis has improved with advances in immunosuppressive therapy, but it remains a leading cause of morbidity in SLE [9]. Early diagnosis and aggressive treatment are crucial to improving renal outcomes and overall survival. In summary, SLE is a complex autoimmune disease with significant renal involvement. Lupus nephritis, particularly when associated with FSGS, poses a considerable challenge in management due to its impact on renal function and patient quality of life. Comprehensive evaluation, including renal biopsy, and tailored therapeutic strategies are essential for optimal patient outcomes.

## Conclusions

In conclusion, the coexistence of FSGS with SLE and lupus nephritis poses a therapeutic challenge. Some patients may not respond to standard treatments, emphasizing the need for a repeat renal biopsy to establish an accurate diagnosis and provide personalized care. A collaborative approach involving rheumatologists and nephrologists is essential to deliver optimal care for patients navigating the complexities of these overlapping conditions, ensuring the best possible outcomes.
